# The Putative Role of Natural Killer Cells in Patients with Hepatitis C Virus-Related Hepatocellular Carcinoma

**DOI:** 10.31557/APJCP.2021.22.8.2559

**Published:** 2021-08

**Authors:** Mona M Hassona, Enas M Radwan, Eman Abdelsameea, Suzanne Estaphan, Heba E Abd Elrhman, Mohamed Abdel-Samiee, Mary Naguib

**Affiliations:** 1 *Department of Clinical and Chemical Pathology, National Liver Institute, Menoufia University, Shebeen El-Kom, Egypt. *; 2 *Department of Clinical Pathology, National Cancer Institute, Cairo University, Cairo, Egypt. *; 3 *Hepatology and Gastroenterology, National Liver Institute, Menoufia University, Shebeen El-Kom, Egypt. *; 4 *Department of Physiology, Faculty of Medicine, Cairo University, Cairo, Egypt. *; 5 *Department of Clinical Pathology, Faculty of Medicine, Zagazig University, Egypt. *

**Keywords:** Hepatocellular carcinoma, hepatitis C Virus, natural killer cells, NKG2D receptor

## Abstract

**Background:**

Natural Killer (NK) cells have crucial roles in immune responses against malignant transformation including hepatocellular carcinoma (HCC). The NKG2D receptor has a critical role in the NK recognition of target cells.

**Aim:**

We assessed NKG2D receptor expression as a diagnostic biomarker for HCC detection and progression in Egyptian patients with hepatitis C virus (HCV)-related HCC.

**Methods:**

We classified 81 patients into three groups: chronic hepatitis (21), cirrhotic (30) and HCC (30) patients, with 36 individuals enrolled to the control group. We analyzed NK levels in peripheral blood and NKG2D receptor expression in NK cells using flow cytometry. Results: We observed a significant decrease in NKG2D (CD314) expression on circulating NK cells and frequency of NK cells expressing NKG2D (CD314) in HCC patients. Also, in patients, larger foci lesions significantly correlated with decreased NK cell numbers. Multiple foci numbers and patients with a Child score C significantly correlated with decreased circulating NK cells expressing NKG2D and decreased NKG2D expression.

**Conclusion:**

The percentage of NK cells in peripheral blood and NKG2D receptor expression could function as potential biomarkers for HCC detection and progression.

## Introduction

Hepatocellular carcinoma (HCC) is a pronounced health challenge and is ranked sixth as the most common global cancer (Forner et al., 2018). However, in Egypt, HCC is the fourth most common cancer (Akinyemiju et al., 2017) which may be attributable to the increased prevalence of hepatitis C virus (HCV) in the country (Abd-Elsalam et al., 2018; Rashed et al., 2020).

Typically, HCC symptoms are not evident, meaning most patients are diagnosed at late stages. As a result, there are few treatment options with limited survival outcomes (Dimitroulis et al., 2017). Thus, the shortage of therapeutic treatment options and HCC resistance to several chemotherapies (Lohitesh et al., 2018) warrants the comprehensive investigation of different HCC pathophysiological pathways to develop viable alternative strategies.

In the tumor microenvironment, anti-tumor immunity is related to infiltrating immune cell crosstalk with each other and with adjacent tumor cells (Balouchi-Anaraki and Nourozian, 2018). Therefore, studying different HCC immuno-landscapes could provide promising therapeutic options.

As the main cell type of the innate immune system, natural killer (NK) cells exert crucial roles in the body’s immune response against the hepatitis B virus (HBV) or HCV infected cells and malignant cells (Juengpanich et al., 2019). NKs eliminate virus-infected cells using direct killing methods (Fathy et al., 2010) via apoptosis (Shabani et al., 2014) or indirectly through cytokine secretion, such as interferon-γ (IFN-γ) and tumor necrosis factor-α (TNF- α) (Wang et al., 2012).

NK cell activation is controlled by the incorporation of signals from various inhibitory and activating receptors (Lanier, 2005). Natural killer group 2D ( NKG2D) is a key activating receptor implicated in the NK recognition of target cells (Molfetta et al., 2017; Zingoni et al., 2018). In essence, these receptors facilitate the NK cells’ cytotoxic function (Juengpanich et al., 2019).

Previous studies reported that NKG2D expression may be unchanged, downregulated or upregulated during HCV infection (Jinushi et al., 2004; De Maria et al., 2007, Ahlenstiel et al., 2010). Activated NKG2D receptor on surface of NK cell can bind to NKG2D ligand expressed in the tumor cells, helping NK cells to activate and kill tumor cells. Through different mechanisms, tumors can escape immune clearance which is mediated by NKG2D receptor/NKG2DL. Expression of *NKG2D* receptor on surface od NK cells can be regulated by molecules, cells and hypoxia in microenvironment of the tumor (Duan et al., 2019). 

To investigate this phenomenon, we assessed NK cell activation status using* NKG2D* receptor expression as a potential diagnostic biomarker for HCC detection and progression, with a view to using it as a possible therapeutic target for HCC in Egyptian patients with chronic HCV infection.

## Materials and Methods

*Study timeline and ethical considerations*

We conducted this study between October 2018 and October 2019 at the National Liver Institute, Menoufia University, Egypt. The study protocol was reviewed and sanctioned by the Institutional Review Board (Ref; IRB00144/2018). The study conformed to the Declaration of Helsinki (1964, revised in 2004). All subjects provided written informed consent.

*Patients and control subjects*

We conducted this study in 81 patients with chronic HCV infection. All were recruited from the Hepatology Department, National Liver Institute, Menoufia University, Egypt. They were categorized into three groups: 1) chronic hepatitis group (21 patients), 2) cirrhotic group (30 patients), and 3) HCC group (30 patients). We also enrolled 36 healthy unrelated subjects as the control group. Patients >75 years or <18 years, co-infected with HBV, and with liver tumors other than HCC were excluded.

All patients with confirmed HCV-Ab positive and detectable HCV-RNA level for >6 months were included in the chronic hepatitis group (group 1). We used clinical, ultrasonographic and laboratory findings to diagnose cirrhotic patients in group 2 (Schuppan and Afdhal, 2008). We also used the Child-Pugh classification system to assess liver cirrhosis severity (Pugh et al., 1973) in this group. Total scores used to determine the grade of Child-Pugh: grade A (5–6 points), grade B (7–9 points) and grade C (10–15 points). HCC was non-histologically diagnosed in the HCC group (group 3) according to American Association for the Study of Liver Disease criteria. We identified the presence of an arterial hyper-vascular focal lesion >2 cm with rapid wash-out with a single imaging modality (magnetic resonance imaging, triphasic spiral computed tomography or angiography) or two imaging modalities demonstrating the before mentioned feature for lesions <2 cm identified HCC (Bruix and Sherman, 2011). 

*Patient and control subject blood biochemistry evaluation*

Routine laboratory tests were performed after complete history taking and complete clinical and radiological examinations. Blood samples were assessed for routine tests: complete blood counts (Sysmex XT-1800i, Sysmex Corporation, Kobe, Japan), liver and renal function tests; alanine aminotransferase (ALT), aspartate aminotransferase (AST), gamma glutamyl transpeptidase (GGT), alkaline phosphatase (ALP), albumin, total bilirubin, direct creatinine, and alpha fetoprotein (AFP) (Cobas 6000, Roche Diagnostics GmbH, Mannheim, Germany). Prothrombin times and international normalized ratios (INR) were assessed using the Sysmex CS-1600 (Sysmex Europe GmbH, Norderstedt, Germany). All patients demonstrated a positive test for the HCV-Ab using third generation enzyme-linked immunosorbent assay, and had detectable HCV-RNA levels using COBAS AmpliPrep/COBAS TaqMan (Roche Diagnostics Ltd, Germany) with detection limit of 15 IU/mL. HBsAg and anti-HBc IgG analyses were performed to exclude co-infection.

*Flow cytometry*

We collected peripheral blood samples from patients and control subjects in ethylenediaminetetraacetic acid tubes and conducted flow cytometric analyses within 24 h. For each sample, 50 μL whole blood was placed in a test tube. After lysis and washing, we added the appropriate volume of each monoclonal antibody (according to manufacturer’s instructions); fluorescein isothiocyanate (FITC)-conjugated anti-human CD3 and phycoerythrin (PE)-conjugated anti-human CD16+CD56 (catalog No. 562365, BD Biosciences), and phycoerythrin cyanin 7 (PE-cy TM 7) conjugated to anti-human NKG2D (CD314) (catalog N. 562365, BD Pharmacogen). We incubated the tubes in the dark for 45 min and then acquired data using a multicolor Navios flow cytometer (Beckman Coulter, Clare, Ireland). Analyses were performed using Kaluza analysis version 2.1 software.

**Figure 1:**
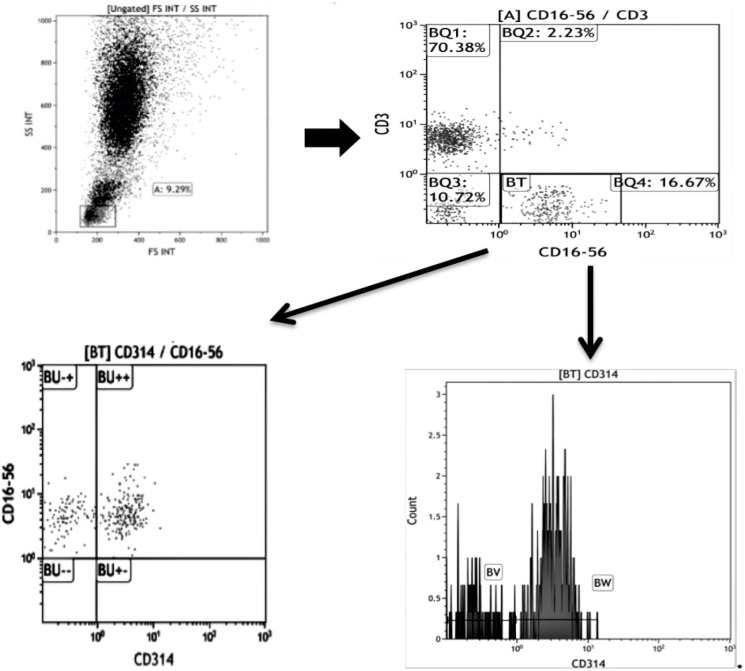
Gating Strategy for NK Cell Detection and Characterization of Phenotypic Expression Pattern for NKG2D (CD314)

Lymphocytes were gated based on their forward scatter versus side scatter properties. NK cells were delineated as lymphocytes with CD16 and CD56 positivity, and CD3 negativity. The percentage of *CD314* expression on NK cells and the mean fluorescence intensity (MFI) of this expression was measured (Figure 1).

*Statistical analysis*

Results were statistically analyzed using the statistical package of social sciences (SPSS 22.0, IBM/SPSS Inc., Chicago, IL, USA). We determined median and interquartile ranges (IQR) to summarize continuous skewed data. Categorical data were expressed as frequencies and percentages. For normally distributed variables, we used ANOVA. When normality and homogeneity assumptions were verified by Shapiro-Wilk test, we used Kruskal-Wallis tests as assumptions were violated. The Mann-Whitney or Student’s t-test was used as appropriate to compare two groups. The Pearson Chi-square (*x*^2^) test was used to compare categorical variables. Multiple pairwise comparisons were adjusted using the Bonferroni post hoc test after significant Kruskal-Wallis or Chi-square tests. Spearman correlation coefficient (rs) was calculated to indicate the strength of association between non-normally distributed numerical variables. For all tests, a p < 0.05 significance value was accepted.

## Results

*Patient characteristics*

Patients recruited to the study were gender and age matched. Ages in control, chronic hepatitis, cirrhotic, and HCC groups were; 53.58 ± 4.05, 54.10 ± 5.13, 55.57 ± 4.76, and 56.57 ± 5.66 years old, respectively. There were no significant differences among groups (p = 0.073) and no significant differences regarding gender distribution among groups (Table 1). When compared with control and chronic hepatitis groups, HCC patients showed significantly increased ALT, AST, ALP, GGT, total bilirubin, INR, and creatinine levels (p ≤ 0.001) and significantly decreased albumin levels (p<0.001). There were no significant differences between the cirrhotic and HCC group; however, GGT levels were significantly elevated in patients with HCC (p = 0.032) (Table 1).

AFP was significantly elevated in the HCC group compared with the control (p ˂ 0.001), chronic hepatitis (p ˂ 0.001), and cirrhotic patient groups (p = 0.034). Also, the cirrhotic patient group showed significantly elevated AFP levels when compared with control (p ˂ 0.001) and chronic hepatitis groups (p = 0.010) (Table 1).

However, significant decreases in white blood cell (WBC) counts and platelets were identified in patients with HCC when compared with control (p ˂ 0.001) and chronic hepatitis groups (p ˂ 0.001). In addition, cirrhotic patients showed decreased WBC and platelet levels when compared with control (p ˂ 0.001) and chronic hepatitis groups (p ˂ 0.001 and p = 0.006, respectively) (Table 2).

*NK cells in peripheral blood*

We observed a significant decrease in NK cell percentages in peripheral blood mononuclear cells (PBMNCs) in patients with HCC when compared with control (p < 0.001) and chronic hepatitis groups (p = 0.002). Also, NK percentage in PBMNCs significantly decreased in cirrhotic patients compared with control (p = 0.012) (Table 2).

**Figure 2:**
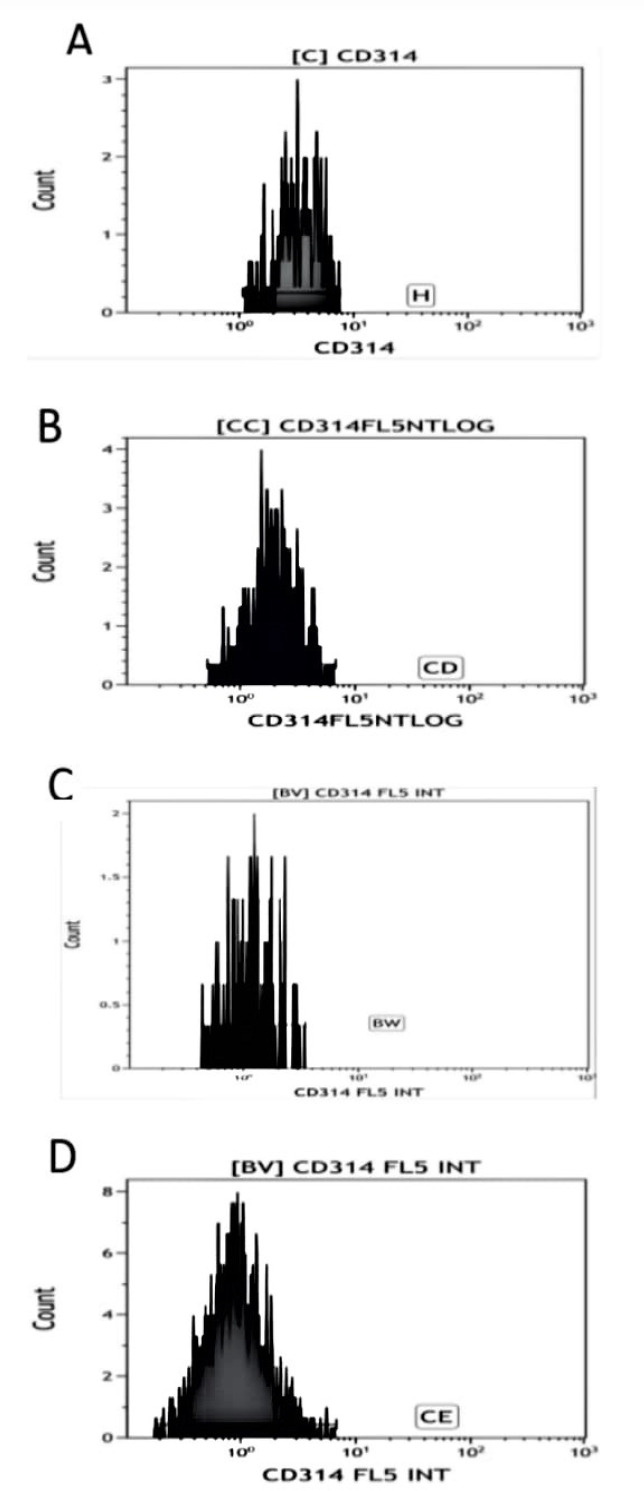
The Mean Florescence Intensity of NKG2D Expression in NK Cells Showing a Progressive Decrease in HCV Positive Patients and with Disease Progression to Cirrhosis then to Controls. (A) control. (B) HCV positive patient. (C) HCV positive patient with liver cirrhosis. (D) HCV positive patient with HCC

In addition, we noted a significant decrease in the percentage of NKG2D expressing NK cells in patients with HCC when compared with control, chronic hepatitis, and cirrhotic patient groups (p < 0.001, p < 0.001, and p = 0.033, respectively). This was associated with a similar decrease in *NKG2D (CD314)* expression in NK cells (p < 0.001, p < 0.001, and p = 0.003, respectively) (Table 2 and Figure 2).

Cirrhotic patients also showed a significant decrease in *NKG2D* expression compared with the control group (p = 0.041). Also, this expression decreased when compared with patients with chronic HCV, but we detected no significance (p = 0.741) (Table 2 and Figure 2).

*Correlation of peripheral NK cells with HCC progression*

We correlated larger foci lesions in the HCC group to decreased total leucocyte counts (p = 0.036). In addition, the percentage of NK cells in PBMNCs decreased significantly in patients with HCC with larger foci tumors (p = 0.008). Also, we found decreased *NKG2D* expression in NK cells and decreased percentages of NK cells expressing NKG2D in HCC group, but without significant correlations (p = 0.422 and p = 0.513, respectively) (Table 3). 

However, multiple foci numbers in the HCC group were significantly correlated with decreased circulating NK cells expressing *NKG2D* (p = 0.047). They decreased expression of the *NKG2D* Mean florescence intensity (MFI). (p = 0.003) without any significant correlation with NK percentage in PBMNCs (p = 0.683) (Table 4).

We noted that patients with HCC with a Child score, C displayed significantly decreased percentages of NK cells expressing NKG2D (p = 0.008) and decreased *NKG2D* expression on NK cells (p = 0.046), with no significant difference in NK percentage in PBMNCs (p= 0.946) (Table 5).

*NKG2D putatively discriminates between cirrhosis and HCC groups*

**Figure 3:**
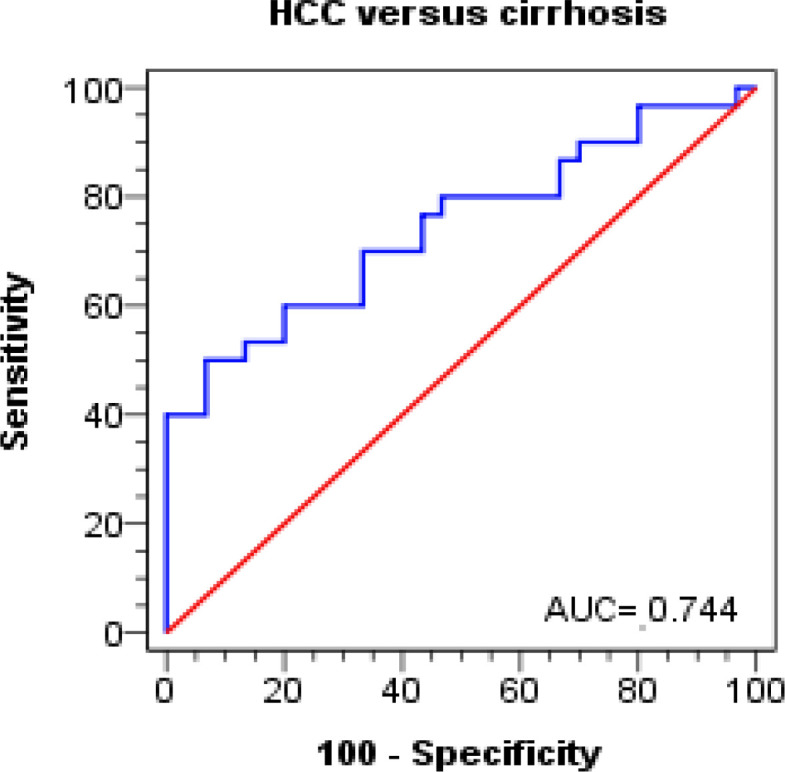
ROC Curves of NKG2D% (CD 314) on NK Cells for Discrimination between Cirrhosis and HCC Groups

**Figure 4:**
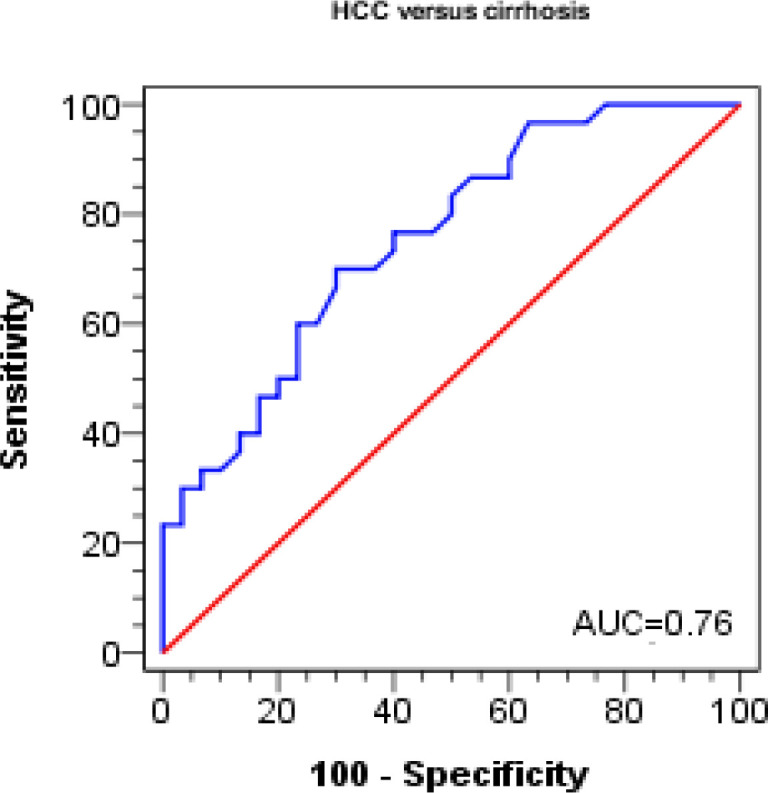
ROC Curves of MFI for Discrimination between Cirrhosis and HCC Groups

Receiver operating curves showed that the percentage of cells expressing *NKG2D* and *NKG2D* expression levels significantly predicted HCC in cirrhotic patients (p = 0.001 and p < 0.001, respectively). A cutoff value of 52.81 or lower for NKG2D percentages on NK cells and 1.47 or lower for NKG2D MFI distinguished patients with HCC from cirrhotic patients, with a sensitivity of 50% and 70%, respectively, a specificity of 93.3% and 70%, respectively, and an accuracy of 71.7% and 70%, respectively (Table 6, Figures 3 and 4).

**Table 1:** Comparisons between Groups Regarding Patient Characteristics and Biochemical Parameters

Parameters	Control (n=36)	Chronic hepatitis (n=21)	Cirrhosis (n=30)	HCC (n=30)	P-value	Multiple comparisons	
Age (years)					0.073 ^a^		
Mean	53.58 ± 4.05	54.10 ± 5.13	55.57 ± 4.76	56.57 ± 5.66			
Gender [n (%)]					0.217 ^b^		
Female	15 (41.7)	6 (28.6)	7 (23.3)	6 (20.0)			
Male	21 (58.3)	15 (71.4)	23 (76.7)	24 (80.0)			
ALT (U/L)					0.001 ^c^	P1=0.006	P4=0.100
Median (IQR)	26.00 (3.75)	54.00 (26.00)	34.00 (40.00)	52.50 (82.00)		P2=1.000	P5=1.000
						P3= 0.013	P6=0.225
AST (U/L)					<0.001 ^c^	P1=0.191	P4=0.048
Median (IQR)	24.50 (1.75)	34.00 (26.00)	72.00 (53.00)	109.00 (116.00)		P2<0.001	P5<0.001
						P3<0.001	P6=0.329
ALP (U/L)					<0.001 ^c^	P1=0.125	P4=0.042
Median (IQR)	61.00 (12.50)	74.00 (45.00)	153.00 (195.00)	160.00 (143.00)		P2<0.001	P5=0.004
						P3<0.001	P6=1.000
GGT (U/L)					<0.001 ^c^	P1=0.075	P4=1.000
Median (IQR)	24.00 (4.75)	29.00 (3.00)	73.00 (93.00)	70.50 (71.00)		P2<0.001	P5=0.002
						P3<0.001	P6=0.032
Total bilirubin (mg/dL)					<0.001 ^c^	P1=0.151	P4=0.017
Median (IQR)	0.61 (0.15)	0.90 (0.34)	1.70 (1.80)	3.55 (3.68)		P2<0.001	P5<0.001
						P3<0.001	P6=0.072
Albumin (g/dL)					<0.001 ^c^	P1=0.015	P4<0.001
Median (IQR)	4.58 (0.27)	4.00 (0.35)	2.60 (0.50)	2.75 (0.50)		P2<0.001	P5=0.001
						P3<0.001	P6=1.000
Creatinine (mg/dL)					0.001 ^c^	P1=1.000	P4=1.000
Median (IQR)	0.80 (0.17)	0.81 (0.11)	0.98 (0.75)	1.22 (0.51)		P2=0.167	P5=0.114
						P3<0.001	P6=0.551
AFP (ng/mL)					<0.001 ^c^	P1=0.069	P4=0.010
Median (IQR)	2.30 (0.63)	4.40 (3.40)	12.00 (14.00)	71.50 (95.00)		P2<0.001	P5<0.001
						P3<0.001	P6=0.034
INR					<0.001 ^c^	P1=0.189	P4<0.001
Median (IQR)	1.03 (0.06)	1.17 (0.24)	1.35 (0.32)	1.39 (0.31)		P2<0.001	P5<0.001
						P3<0.001	P6=1.000

ALT, Alanine aminotransferase; AST, Aspartate aminotransferase; ALP, Alkaline phosphatase; GGT, Gamma glutamyl transpeptidase; AFP, Alpha fetoprotein; INR, International normalized ratio; Significance level was < 0.05; IQR, Interquartile range (difference between 1st and 3rd quartiles) ;

a, ANOVA test; b, Person Chi-square test; c, Kruskal-Wallis tests; if significant; then multiple pairwise comparisons adjusted by Bonferroni post hoc test; P1, comparison between control and HCV groups; P2, comparison between control and cirrhosis groups; P3, comparison between control and HCC groups; P4, comparison between HCV and cirrhosis groups; P5, comparison between HCV and HCC groups; P6, comparison between cirrhosis and HCC groups

**Table 2:** Comparison between Groups Regarding Immunohematological Parameters

Parameters	Control (n=36)	Chronic hepatitis (n=21)	Cirrhosis (n=30)	HCC (n=30)	P-value ^a^	Multiple comparisons	
WBCs (10^3^ cell/µL)					<0.001	P1=1.000	P4<0.001
Median (IQR)	6.85 (2.05)	7.30 (3.15)	4.65 (1.63)	4.70 (1.53)		P2<0.001	P5<0.001
Range (Min-Max)	4.50 - 9.50	4.30 - 12.30	3.10 - 8.90	3.30 - 8.00		P3<0.001	P6=1.000
MNCs%					<0.001	P1=1.000	P4<0.001
Median (IQR)	32.80 (6.58)	34.60 (11.30)	23.85 (9.23)	19.60 (15.15)		P2=0.001	P5<0.001
Range (Min-Max)	23.60 - 40.00	25.40 - 50.60	15.40 - 44.00	13.20 - 43.00		P3<0.001	P6=1.000
NK% in WBCs					<0.001	P1=1.000	P4=0.016
Median (IQR)	4.38 (1.63)	3.90 (4.05)	1.99 (1.75)	1.24 (2.02)		P2<0.001	P5<0.001
Range (Min-Max)	1.82 - 7.19	1.43 - 8.73	0.78 - 7.94	0.58 - 6.63		P3<0.001	P6=0.694
NK% in MNCs					<0.001	P1=1.000	P4=0.360
Median (IQR)	13.60 (4.49)	11.52 (7.36)	10.01 (8.21)	6.69 (5.98)		P2=0.012	P5=0.002
Range (Min-Max)	7.68 - 19.90	6.14 - 22.11	3.63 - 20.35	2.45 - 17.49		P3<0.001	P6=0.333
NKG2D (CD 314) %					<0.001	P1=1.000	P4=1.000
Median (IQR)	81.34 (13.17)	79.66 (20.54)	73.91 (21.59)	54.01 (31.73)		P2=0.053	P5<0.001
Range (Min-Max)	55.91 - 97.60	51.05 - 95.20	46.96 - 94.00	23.41 - 93.72		P3<0.001	P6=0.033
NKD2D MFI					<0.001	P1=1.000	P4=0.741
Median (IQR)	2.02 (0.71)	1.82 (1.10)	1.73 (0.84)	1.17 (0.80)		P2=0.041	P5<0.001
Range (Min-Max)	1.36 - 3.40	1.05 - 3.41	0.79 - 2.77	0.63 - 2.10		P3<0.001	P6=0.003
Platelets (103 cell/µL)					<0.001	P1=0.015	P4=0.006
Median (IQR)	323.50 (42.00)	212.00 (82.00)	132.00 (49.50)	73.50 (69.75)		P2<0.001	P5<0.001
Range (Min-Max)	264.00 - 392.00	146.00 - 359.00	47.00 - 244.00	50.00 - 200.00		P3<0.001	P6=0.914

WBCs, white blood cells; MNCs, mononuclear cells; NK, Natural killer; MFI, Mean florescence intensity; Significance level was <0.05; IQR, Interquartile range (difference between 1st and 3rd quartiles); ^a^, Kruskal-Wallis test, if significant, then multiple pairwise comparisons adjusted by Bonferroni post hoc test; P1, comparison between control and HCV groups; P2, comparison between control and cirrhosis groups; P3, comparison between control and HCC groups; P4, comparison between HCV and cirrhosis groups; P5, comparison between HCV and HCC groups; P6, comparison between cirrhosis and HCC groups

**Table 3:** Relationships between Tumor Foci Size and Immunohematological Parameters in the HCC Group

Parameters	Foci size		P-value
	Small (< 5 cm) (n=12)	Large (≥ 5 cm) (n=18)	
Age (years)			0.202 ^a^
Median (IQR)	59.50 (9.75)	54.50 (3.00)	
Range (Min-Max)	45.00 - 64.00	47.00 - 68.00	
WBCs (10^3^ cell/µL)			0.036 ^a^
Median (IQR)	5.20 (1.15)	4.15 (1.63)	
Range (Min-Max)	4.20 - 6.70	3.30 - 8.00	
MNCs%			0.001 ^b^
Mean ± SD	30.68 ± 9.26	18.89 ± 4.10	
Range (Min-Max)	3.10 - 41.00	2.30 - 37.90	
NK% in WBCs			0.001 ^a^
Median (IQR)	3.21 (2.90)	0.93 (0.74)	
Range (Min-Max)	0.77 - 6.63	0.58 - 2.25	
NK% in MNC			0.008 ^a^
Median (IQR)	9.05 (7.14)	5.26 (4.16)	
Range (Min-Max)	5.14 - 17.49	2.45 - 14.08	
NKG2D (CD 314) %			0.513 ^b^
Mean ± SD	57.63 ± 18.93	52.67 ± 20.76	
Range (Min-Max)	27.60 - 86.57	23.41 - 93.72	
NKG2D MFI			
Mean ± SD	1.31 ± 0.40	1.17 ± 0.47	0.422 ^b^
Range (Min-Max)	0.74 - 1.91	0.63 - 2.10	

WBCs, white blood cells; NK, Natural killer; MNCs, mononuclear cells; MFI, Mean florescence intensity; IQR, Interquartile range (difference between 1st and 3rd quartiles); SD, standard deviation; Significance level was at < 0.05; ^a^, Mann-Whitney test; ^b^, Student t-test

**Table 4:** Relationship between Foci Number and Immunohematological Parameters in the HCC Group

Parameters	Foci number		P-value
	Single (n=19)	Multiple (n=11)	
Age (years)			0.884 ^a^
Mean ± SD	56.68 ± 6.34	56.36 ± 4.52	
Range (Min-Max)	45.00 - 68.00	50.00 - 64.00	
WBCs (103 cell/µL)			0.155 ^b^
Median (IQR)	4.30 (2.50)	5.10 (0.90)	
Range (Min-Max)	3.30 - 8.00	4.00 - 5.90	
MNCs%			0.451 ^b^
Median (IQR)	19.30 (18.60)	23.60 (13.60)	
Range (Min-Max)	13.20 - 43.00	16.10 - 35.60	
NK% in WBCs			0.931 ^b^
Median (IQR)	1.41 (1.96)	1.13 (2.45)	
Range (Min-Max)	0.58 - 6.63	0.58 - 4.53	
NK% in MNC			0.683 ^b^
Median (IQR)	6.66 (7.09)	6.72 (7.20)	
Range (Min-Max)	3.18 - 17.49	2.45 - 14.45	
NKG2D (CD 314) %			0.047^ a^
Mean ± SD	60.07 ± 19.25	45.29 ± 18.04	
Range (Min-Max)	27.60 - 93.72	23.41 - 71.55	
NKG2D MFI			0.003^ a^
Mean ± SD	1.38 ± 0.46	0.96 ± 0.24	
Range (Min-Max)	0.63 - 2.10	0.67 - 1.33	

WBCs, white blood cells; NK, Natural killer; MNCs, mononuclear cells; MFI, Mean florescence intensity; Significance level was at< 0.05; IQR, Interquartile range (difference between 1st and 3rd quartiles); SD, standard deviation; ^a^, Student t-test ; ^b^, Mann-Whitney test

**Table 5:** Relationship between Child-Pugh Score (Class) and Immunohematological Parameters in the HCC Group

Parameters	Child Class		P-value
	Child B (n=21)	Child C (n=9)	
Age (years)			0.097 ^a^
Median (IQR)	58.00 (11.00)	54.00 (3.00)	
Range (Min-Max)	45.00 - 68.00	50.00 - 56.00	
WBCs (103 cell/µL)			0.107 ^b^
Mean ± SD	5.07 ± 1.32	4.47 ± 0.64	
Range (Min-Max)	3.30 - 8.00	3.50 - 5.60	
MNCs%			0.099 ^a^
Median (IQR)	21.20 (17.50)	17.70 (9.55)	
Range (Min-Max)	14.40 - 43.00	13.20 - 28.60	
NK% in WBCs			0.442 ^a^
Median (IQR)	1.17 (2.75)	1.31 (0.95)	
Range (Min-Max)	0.58 - 6.63	0.58 - 2.25	
NK% in MNCs			0.946^ a^
Median (IQR)	6.22 (4.93)	7.39 (9.39)	
Range (Min-Max)	3.18 - 17.49	2.45 - 14.08	
NKG2D (CD 314) %			0.008^ b^
Mean ± SD	60.72 ± 19.69	40.49 ± 11.82	
Range (Min-Max)	27.60 - 93.72	23.41 - 55.75	
NKG2D MFI			
Median (IQR)	1.33 (0.84)	1.10 (0.51)	0.046^ a^
Range (Min-Max)	0.63 - 2.10	0.67 - 1.33	

WBCs, white blood cells; NK, Natural killer; MNCs, mononuclear cells; MFI, Mean florescence intensity; Significance level was at< 0.05; IQR, Interquartile range (difference between 1st and 3rd quartiles); SD, standard deviation; ^a^, Mann-Whitney test; ^b^, Student t-test

**Table 6:** Diagnostic Performance of NKG2D% (CD 314) for Discrimination between Cirrhosis and HCC Groups

Test characteristics	HCC versus cirrhosis	
	NKG2D% (CD 314)	MFI
Best cutoff value	≤ 52.81	≤ 1.47
AUC	0.74	0.76
P-value	0.001	<0.001
Sensitivity %	50	70
Specificity %	93.3	70
PPV %	88.2	70
NPV %	65.1	70
Accuracy %	71.7	70

PPV, Positive predictive value; NPV, Negative predictive value; Significance level was at < 0.05

## Discussion

Several studies reported the effects of immuno-surveillance during HCC progression (Foerster et al., 2018; Inada et al., 2019). As an important cell component in innate immunity, NKs are essential for attacking and neutralizing microorganisms and eliminating aberrantly transformed cells (Vivier et al., 2008). Nevertheless, these roles require further investigation, especially those played by NKs in chronic HCV infection.

NK cells comprise 5%–20% of PBMNCs. Based on CD56 density expression (adhesion molecule) and the ADCC-mediating FcγRIIIA receptor, CD16, we divided NKs into different populations (Caligiuri, 2008; Angelo et al., 2015; Amand et al., 2017). Our study used combined anti-CD16 and anti-CD56 antibodies conjugated to the same fluorochrome to derive a broad recognition of NK cells.

We observed that NK cell percentages in PBMNCs decreased in chronic HCV and cirrhotic patients compared to controls.

Previous studies also reported decreased NK cells in the peripheral blood of patients with chronic hepatitis C compared with healthy controls (Meier et al., 2005; Rafik et al., 2012; El Deeb et al., 2013). Previously, some studies anticipated this decreased NK frequency to recruit NK cells in the liver with chronic hepatitis under the effect of chemokines and cytokines secreted by Kupfer cells, liver sinusoidal endothelial cells, and T cells (Grégoire et al., 2007).

It is worth noting that NK cells are normally represented at 30%–50% in the liver, establishing them as major intrahepatic immune cells (Doherty and O’Farrelly, 2000; Gao et al., 2009). However, Bonorino et al., (2009) and Tatsumi and Takehara (2016) indicated that reduced NK cells in the liver were associated with decreased NKs in the peripheral blood. Bonorino et al., (2009) also reported that chronic HBV infection was not associated with an NK reduction suggesting the role of chronic HCV infection.

We also observed that the percentage of NK cells in PBMNCs decreased in the HCC group when compared with the control group, in agreement with Zhou (2010). This could be due to changes in cytokine levels (Juengpanich et al., 2019).

NK cell receptors exert critical roles in controlling NK cell responses. Their dysregulated expression may be implicated in HCV infection chronicity due to the cellular immune responses’ ineffectiveness (Nattermann et al., 2006).

In this study, we detected reduced expression of the NKG2D Mean florescence intensity (MFI).with decreased frequency of NK cells expressing CD314 (NKG2D) in HCC group. 

Chronic inflammation provoked by persistent HCV infection could reduce NK cell functions, which may affect the liver’s progression to HCC (Sun et al., 2015). Different viruses and malignant cells have established different mechanisms to escape NKG2D-mediated recognition, thereby emphasizing the significance of the NKG2D defense system (Stern-Ginossar et al., 2007; Jonjic et al., 2008).

Also, Cariani et al., (2016) reported reduced frequencies of NK cells carrying the activated NKG2D receptor in patients with HCV-related HCC compared to healthy controls. In addition, these authors reported decreased *NKG2D* expression in patients with HCC.

Zekri et al., (2018) indicated that levels of active NK cells expressing NKG2D were statistically decreased in chronic hepatitis, cirrhotic, and HCC groups when compared with controls. This phenomenon was associated with increased interleukin-10 (IL-10) levels in cirrhotic and HCC groups compared to controls, which could account for this tolerant state. Also, decreases in IFN-γ levels in chronic HCV, cirrhosis, and HCC patients compared with control could explain poor cytotoxicity.

NK receptor ligands are chief evasion foci from immune responses. Formerly, malignant cells discharge soluble MICA (sMICA) forms into serum, interfering with MICA/NKG2D- stimulating signals (Groh et al., 2002). Furthermore, sMICA levels increased at later HCC stages, which we associated with the reduced activation of NK cells associated with *NKG2D* expression downregulation (Jinushi et al., 2005). Also, NKG2D ligand shedding by tumor cells reduces ligand density on the cell surface, thus reducing a predisposition to NKG2D-mediated cytotoxicity (Salih et al., 2008).

In addition, increased *AGP *expression in HCC cells may ultimately reduce the IL-12 production from dendritic cells, resulting in decreased *NKG2D* expression and eventually reducing NK cell activation (Yamamoto et al., 2011).

Also, direct contact with HCV-infected cells may impair NK cell cytotoxicity and IFN-γ production via NKG2D and NKp30 downregulation on NK cells (Yoon et al., 2011).

Oliviero et al., (2009) reported that circulating NK cells were significantly decreased in patients with chronic HCV than controls. Yet, there was bias to activation phenotype of NK cells in HCV patients manifested by significantly increased frequency of NK cells expressing activating receptor NKG2D with a simultaneous decrease in NK cells expressing inhibitory receptors. This could be explained by racial and environmental differences with different viral genotypes and different clinical stages in study patients.

Sène et al., (2010) reported no significant difference in circulating NK cell percentage between patients with chronic HCV and controls; however, they noted inhibited *NKG2D* expression and impaired NK functions. They also demonstrated that HCV-NS5A led to IL-10 secretion from monocytes, followed by increased transforming growth factor-β (TGF- β) production, which could be responsible for NK inhibition.

The controversy concerning NK frequency in the peripheral blood and different NK phenotypic characteristics may be attributable to various study factors; patients with different viral loads, HCV genotypes, different detection methods, different disease stages, or variable populations.

Furthermore, we investigated the effects of peripheral NKs on hepatocarcinogenesis progression. We noticed that larger foci lesions in the HCC group significantly correlated with decreased NK cells in PBMNCs. Multiple foci numbers and patients with Child score C in the HCC group were significantly correlated with decreased circulating NK cells expressing *NKG2D* and decreased expression of the activation marker. 

Moreover, the percentage of cells expressing *NKG2D *and NKG2D MFI significantly characterized the presence of HCC in cirrhotic patients, with cutoff values of 52.81 or lower and 1.47 or lower, respectively.

In conclusion, the various strategies performed by HCV to evade the surveillance of NKG2D pathway immune responses represent promising targets for immunotherapy. Measuring NK levels in the peripheral blood and detecting NKG2D receptor expression on NK cells could provide possible biomarkers for HCC detection and progression. These observations should be confirmed by further comprehensive studies with larger sample sizes.

## Author Contribution Statement

None. 
